# Remarks on the Dysentery, with an Appendix on Putrid Fevers

**Published:** 1784

**Authors:** 


IV. *Betrachtungen über die Ruhr, &c.* i. e. *Remarks on the Dysentery, with an appendix on putrid fevers. By* Christian Lewis Mursinna, *surgeon major of the regiment of Petersdorf.* 8vo. Berlin, 1780. 140 pages.

THE dysentery which prevailed at Herford in Westphalia, and which is the principal subject of this little work, first made its appearance about the close of July 1779. During the first week or two it was confined chiefly to the lower class of people, who are uncleanly, and badly fed and cloathed; but towards the latter end of August it spread among all ranks of people. The town contains about 3000 inhabitants, and, of these, 340 were labouring under the disease at the beginning of September, when the epidemic is said to have been at its height. Its violence abated as the heat of the weather subsided, and before the beginning of October, we are told, it had almost entirely disappeared.

To oppose the progress of this contagion, a council of health, composed of the magistrates, clergy, and physicians of the town, gave directions for the interment of the dead on the day on which they died, in a place at some distance

 from from the town. At the ſame time the town was divided into ſixteen diſtricts, in each of which two perſons of known vigilance and probity were appointed to enforce the precautions and modes of treatment recommended by the phyſicians. To theſe circumſtances and the liberality with which the poor were aſſiſted by the more opulent inhabitants, our author thinks it was in a great meaſure owing, that of 660 patients who were attacked with the dyſentery in the courſe of two months, only 168 died.

Among the prediſpoſing cauſes of this epidemic our author enumerates the uncommon heat of the ſummer; the occupation of the greater number of the inhabitants, who are employed in agriculture; their food, which conſiſts chiefly of ſalted and ſmoaked meat; their want of cleanlineſs; the ſituation of the town, which is marſhy; and laſtly, neglect at the firſt attack of the diſeaſe.

He remarks, that patients in affluent circumſtances had commonly only ſlight attacks of the diſeaſe, and ſometimes ſimply a diarrhœa. He had occaſion to obſerve that fear, in ſome caſes, ſeemed to aſſiſt in propagating the diſeaſe.

This dyſentery, we are told, was commonly ſudden in its attack. It uſually began with a ſhivering, vering, and pain in the loins; to theſe ſucceeded violent pains in the belly, and frequent ſtools, which were oftentimes bloody and always mucous. The greater number of patients preſerved their appetite for the firſt day or two, but in a few the ſtomach was diſordered even at the beginning. The pulſe was ſeldom affected till the diſeaſe had made ſome progreſs, and then it often became extremely quick. The treatment which was found to be moſt ſucceſsful conſiſted in the uſe of emetic tartar, eighteen grains of which were diſſolved in ſix ounces of water ſweetened with ſyrup. Of this mixture a table ſpoonful was directed to be taken every half hour till the patient vomited. To this was added the uſe of a purging mixture of two ounces of tamarinds, and half an ounce of Glauber's ſalt, diſſolved in four ounces and a half of water, and ſweetened with an ounce of ſyrup: of this a tea cupful was adminiſtered every two hours. In ſome caſes, where the ſtate of the pulſe ſeemed to indicate it, bleeding was had recourſe to, and when the pains were violent bliſters were ſometimes applied with ſucceſs to the belly, and to the calves of the legs. Glyſters were likewiſe frequently thrown up, and fomentations to the belly, of linſeed oil, camphor and opium, were found to be uſeful in mitigating pain.

